# L-DOPA Uptake in Astrocytic Endfeet Enwrapping Blood Vessels in Rat Brain

**DOI:** 10.1155/2012/321406

**Published:** 2012-07-24

**Authors:** M. Y. Inyushin, A. Huertas, Y. V. Kucheryavykh, L. Y. Kucheryavykh, V. Tsydzik, P. Sanabria, M. J. Eaton, S. N. Skatchkov, L. V. Rojas, W. D. Wessinger

**Affiliations:** ^1^Department of Physiology, Universidad Central del Caribe, Bayamón, PR 00956, USA; ^2^Department of Pharmacology and Toxicology, University of Arkansas for Medical Sciences, Little Rock, AR 72205, USA

## Abstract

Astrocyte endfeet surround brain blood vessels and can play a role in the delivery of therapeutic drugs for Parkinson's disease. However, there is no previous evidence of the presence of LAT transporter for L-DOPA in brain astrocytes except in culture. Using systemic L-DOPA administration and a combination of patch clamp, histochemistry and confocal microscopy we found that L-DOPA is accumulated mainly in astrocyte cell bodies, astrocytic endfeet surrounding blood vessels, and pericytes. In brain slices: (1) astrocytes were exposed to ASP^+^, a fluorescent monoamine analog of MPP^+^; (2) ASP^+^ taken up by astrocytes was colocalized with L-DOPA fluorescence in (3) glial somata and in the endfeet attached to blood vessels; (4) these astrocytes have an electrogenic transporter current elicited by ASP^+^, but intriguingly not by L-DOPA, suggesting a different pathway for monoamines and L-DOPA via astrocytic membrane. (5) The pattern of monoamine oxidase (MAO type B) allocation in pericytes and astrocytic endfeet was similar to that of L-DOPA accumulation. We conclude that astrocytes control L-DOPA uptake and metabolism and, therefore, may play a key role in regulating brain dopamine level during dopamine-associated diseases. These data also suggest that different transporter mechanisms may exist for monoamines and L-DOPA.

## 1. Introduction

 It is a widely held opinion that the transport systems at the blood-brain barrier (BBB) are localized in the brain capillary endothelial cells and control the exchange of various endogenous and exogenous compounds between the circulating blood and brain. Astrocytes, the glial cells that compose the gliovascular interface [1], are an important part of the brain vascular system. Astrocytic endfeet wrap blood vessels, forming the second barrier around the endothelial cells that is separated from endothelium by a space filled with basal lamina. Basal lamina, a gel that consists of laminin, fibronectin, tenascin, collagens, and proteoglycan [2], separates the astrocytic endfeet and endothelium cell layers but does not prevent passage of macromolecules. The ~20 nm gap between adjacent astrocytic endfeet, which is diffusible by horseradish peroxidase [3], sometimes gives rise to questions about the ability of astrocytes to physically contribute to the BBB; hence, the barrier and transport roles of astrocytes have been underappreciated. Nevertheless, astrocytes participate in the transport of substrates to the brain [4] and possess a variety of transport systems that play roles in the delivery of therapeutic drugs for Parkinson's disease (PD). Tyrosine, a precursor of dopamine, and l-DOPA are extracted from the circulating blood by the amino acid transport system l, involving l-type amino acid transporter 1 (LAT1)/4F2 heavy chain (4F2hc) complex [5–9]. This transporter has previously been identified and characterized in cultured astrocytes [10, 11]. Additionally, variants of the organic cation transporter 1, also previously identified in cultured astrocytes [12, 13], may also be involved in transport of l-DOPA [14, 15]. These observations led us to ask whether astrocytes participate in l-DOPA transport in the brain as well.

 Early whole animal studies showed that after an injection of l-DOPA to the circulation, l-DOPA or its products accumulated in brain capillary endothelium and in pericytes [16–18]. Here, using rat brain slices and confocal microscopy, we report the accumulation of l-DOPA in astrocyte cell bodies and in the endfeet surrounding blood vessels. Also, the distribution pattern of MAO type B in pericytes and astrocytic endfeet was similar to the l-DOPA accumulation pattern.

## 2. Materials and Methods

### 2.1. Histochemistry

 A modified Falck-Hillarp method was used to visualize l-DOPA uptake in slices [19]. Briefly, slices were incubated in freshly made glyoxylate-containing solution at 4°C for 2 h, placed on a glass slide, dried with air blower at room temperature for 1.5–2 h, heated in an oven at 85°C for 9 min, covered with paraffin oil, coverslipped, and viewed using a Fluoview FV1000 fluorescence confocal microscope (Olympus, Japan) with UV-GFP filter set. The incubating solution contained (in mM) 500 sodium glyoxylate, 40 *N*-2-hydroxyethylpiperazine-*N*′-2-ethanesulfonic acid (HEPES), and 100 sucrose, dissolved in deionized water, pH 7.0. The final pH of the solution was adjusted to 7.0 with either glyoxylic acid or sodium bicarbonate crystals. A FITC filter set was used to reveal ASP^+^ staining.

To visualize MAO-B, we used diaminobenzidine as a chromogen and tyramine as a substrate according to the protocol of Ryder et al. [20] as modified by Willoughby et al. [21]. During this reaction, we blocked MAO-A using the irreversible MAO-A-selective inhibitor *N*-[3-(2,4-dichlorophenoxy)propyl]-*N*-methyl-prop-2-yn-1-amine (clorgyline).

### 2.2. Animals and Slice Preparation

All experimental procedures were performed in accordance with the US Public Health Service Publication Guide for the Care and Use of Laboratory Animals and were approved by the Animal Care and Use Committee at the Universidad Central del Caribe. Sprague-Dawley rats of either sex between 20 and 30 days of age were decapitated. Hippocampal slices (200 *μ*M) were prepared using a vibratome (VT1000S, Leica Microsystems GmbH, Wetzlar, Germany) in artificial cerebrospinal fluid (ACSF) containing (in mM) 127 NaCl, 2.5 KCl, 1.25 NaH_2_PO_4_, 25 NaHCO_3_, 2 CaCl_2_, 1 MgCl_2_, and 25 d-glucose, ice-cold, saturated with a 95% O_2_-5% CO_2_ gas mixture at pH = 7.4. Slices were perfused with the same ACSF at room temperature. BaCl_2_ (100 *μ*M) was added to ACSF for the perfusion.

 For systemic injections we used 60-day-old Sprague-Dawley rats. Intravenous injections of l-DOPA (2 mM) or ASP^+^ (0.5 mM) in a 0.5 mL volume were made via the lateral tail vein.

### 2.3. Whole Cell Recordings

 Membrane currents were measured with the single-electrode whole-cell patch-clamp technique. Cells were visualized using an Olympus infrared microscope equipped with DIC (BX51WI Olympus, Japan). Two piezoelectric micromanipulators (MX7500 with MC-1000 drive, Siskiyou, Inc., Grants Pass, OR) were used for voltage-clamp and current-clamp recording and for positioning a micropipette with a 30–50 *μ*m tip diameter for application of test solutions. A MultiClamp 700A patch-clamp amplifier with a DigiData 1322A interface (Molecular Devices, Inc., Sunnyvale, CA) was used for recording and stimulation. The pClamp 10 software package (Molecular Devices, Inc., CA) was used for online data acquisition and analysis. Borosilicate glass pipettes (O.D. 1.5 mm, I.D. 1.0 mm; World Precision Instruments, Sarasota, FL) were pulled to a final resistance of 8–10 MΩ for astrocyte recordings in four steps using a P-97 puller (Sutter Instrument Co., Novato, CA). Electrodes were filled with the following solution (in mM): 130 K-gluconate, 10 Na-gluconate, 4 NaCl, 4 phosphocreatine, 0.3 GTP-Na_2_, 4 Mg-ATP, and 10 HEPES, and the pH was adjusted to 7.2 adjusted with KOH. Astrocyte recordings were considered only if membrane potential was negative to –75 mV and if cells had linear current voltage relation (variably rectifying astrocytes according to [22]) and low input resistance (less than 20 MΩ). Pipette potential was not corrected.

### 2.4. Materials

4-[4-(dimethylamino)-styryl)-*N*-methylpyridinium] (ASP^+^), 1,1′-diethyl-2,2′-cyanine iodide (decynium-22), sodium glyoxylate, *N*-2-hydroxyethylpiperazine-*N*′-2-ethanesulfonic acid (HEPES), glyoxylic acid and other chemicals were purchased from Sigma-Aldrich Corp. (St. Louis, MO).

## 3. Results

### 3.1. Uptake of **l-DOPA** and ASP^+^ after Intravenous Injection

l-DOPA is extracted from the circulating blood by LAT1 transport system, which is found in astrocytes [10, 11]. It was shown previously that l-DOPA can pass through the blood-brain barrier and for a short time (15–60 sec) is accumulated in blood vessel walls and pericytes, partially being decarboxylated to dopamine [16–18]. Since the astrocytic endfeet processes wrap blood vessels, we expected astrocyte involvement as well after systemic injection of l-DOPA. After a 2 mM intravenous injection of l-DOPA we waited 30 sec, then we decapitated the animal, removed the brain, and dissected the hippocampal area out. This tissue was fixed for 30 sec in 4% paraformaldehyde, and then 200 *μ*m hippocampal slices were prepared and processed using Falck-Hillarp method [19].

 Similar experiments were conducted after intravenous injections of 0.5 mM ASP^+^. ASP^+^ is a known fluorescent substrate for high-affinity monoamine transporters and organic cation transporters [23–25].

**Figure 1:**
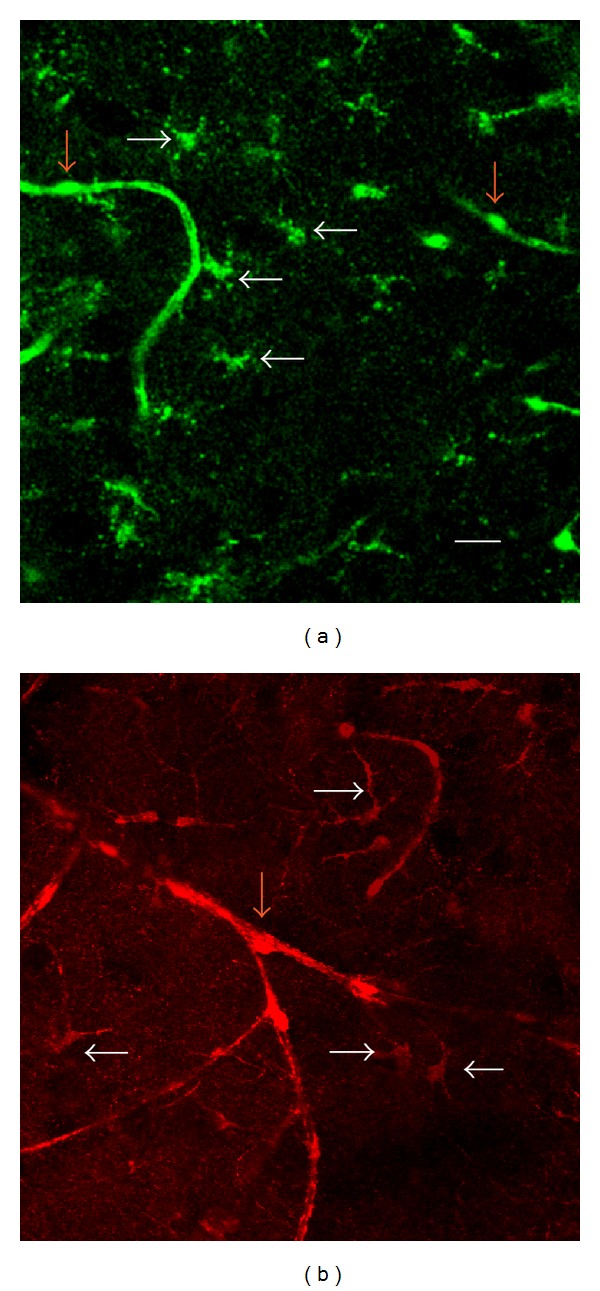
Uptake in rat hippocampus following an intravenous injection of l-DOPA (a) or ASP^+^ (b). Red arrows: pericytes, white arrows: presumable astrocytes, identified by morphology. Scale for (a) and (b): 20 *μ*m.

 After either l-DOPA or ASP^+^ injections, fluorescence was clearly visible from cells identified by appearance as astrocytes, sending their processes to the walls of blood vessels (Figures 1(a) and 1(b), presumable astrocytes marked with white arrows). The intensive uptake of l-DOPA by pericytes has been described previously [16, 17], and they were also visible (Figure 1, pericytes marked by red arrows). Some reduced fluorescence was also visible in capillary endothelium, corresponding to Wade and Katzman's [17] results showing endothelium l-DOPA fluorescence that peaked at 15 sec after injection and was significantly reduced 2 min later.

### 3.2. Electrophysiological Characterization of Astrocytes and ASP^+^ Uptake in Slices

**Figure 2:**
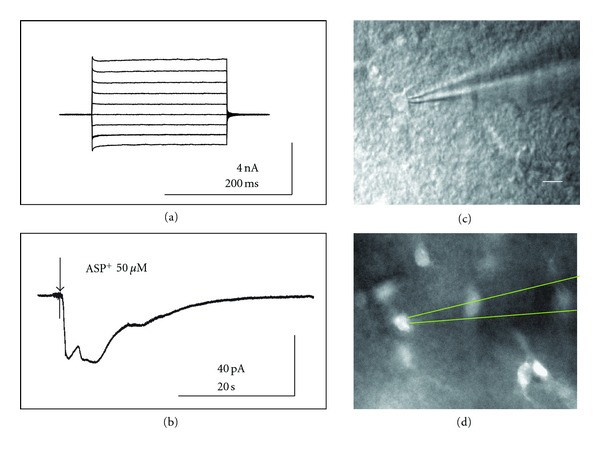
Accumulation of ASP^+^, the fluorescent substrate for high-affinity monoamine transporters and organic cation transporters in astrocytes in hippocampal brain slices. (a) Astrocytes current response to voltage step protocol application reveals a linear IV-relationship. (b) Puff-application of ASP^+^ elicited transporter current in astrocytes, maintained previously at zero current. Arrow indicates the moment of application. (c) and (d) The same astrocyte with attached patch-pipette before (in (c), infrared DIC) and after (in (d), fluorescence) the application of 50 *μ*M ASP^+^. Scale on (c) and (d) 20 *μ*m.

 In order to confirm that the cells that we identified after the fluorescent staining as astrocytes by their shape and the size were really astrocytes, we used brain slices for electrophysiological characterization and ASP^+^ uptake studies. In these experiments we were interested to determine if ASP^+^ was taken up by astrocytes in brain slices. For this purpose, astrocytes in stratum radiatum area of hippocampus were first electrophysiologically characterized using whole-cell voltage-clamp; to be sure we are working with passive (linear) astrocytes. We studied astrocytes that were 30–50 *μ*m deep in the slice, cells (with cell body ~10 *μ*m) were identified first under infrared DIC optics (Figure 2(c)). After a standard patching procedure, a voltage step protocol was applied, as described by [22]. Only data from cells showing a linear I–V curve (Figure 2(a)) were considered for consistency. The mean membrane potential of astrocytes was –85.2 ± 3.1 mV (*n* = 126), and the mean input resistance was 16.3 ± 2.1 M Ω (*n* = 126).

 Ba^++^ (100 *μ*M) was added to the solution after the membrane potential measurements to reduce the potassium current, which can interfere with the current from the electrogenic transporter. Other known blockers of potassium channels were not suitable for these experiments because they are either substrates (Cs^+^) or nontransportable blockers (quinine) of organic cation transporters [26]. Organic cation transporters are among the possible candidates for l-DOPA and ASP^+^ transport, as they were found in astrocytes [12] and have been shown to transport both of these substrates [14, 24, 25].

 Astrocytes were voltage-clamped at their membrane potential to get zero current. 50 *μ*M ASP^+^ was applied to the astrocyte from a distance of 100 *μ*M, using a puff-electrode with a 4 *μ*M tip and a 0.5 sec pressure injection (Figure 2(b), the moment of the application marked with the arrow). An ASP^+^-elicited inward current was recorded for each clamped astrocyte, and the mean current value at maximum was 47 ± 14 pA (*n* = 126). The current varied from 120 pA to 15 pA in individual astrocytes in different animals. Transport of substrates by organic cation transporters similarly to many other monoamine transporters is known to be electrogenic [26], and thus the current we had recorded in the presence of potassium channel blocker probably represents a transporter current. After the puff-application of ASP^+^, fluorescence was visualized using a CY3 filter set. Figure 2(d) shows that ASP^+^ was taken up by the majority of astrocytes in the slice, including the astrocyte that was connected to the electrode.

 In separate experiments we showed that 2 *μ*M decynium-22, a well-known nonneuronal monoamine transporter blocker [12, 25], reduced the ASP^+^-elicited current in astrocytes by 56.3 ± 14% (*n* = 12), if applied 30 sec prior to the puff application of 50 *μ*M ASP^+^ (data not shown). These data confirm that astrocytes in the hippocampal brain slice, which were clearly identified by electrophysiological methods as passive astrocytes, have effective mechanisms to take up ASP^+^. The relatively large variation of transporter current amplitudes from astrocytes in the same brain area suggests a wide dispersion of transporter quantity in different animals, and possibly a wide range of physiological regulation of the transporter.

### 3.3. **l-DOPA** and ASP^+^ Uptake in Astrocytes in Slices

#### 3.3.1. Double Staining for **l-DOPA** and ASP^+^ Uptake

**Figure 3:**
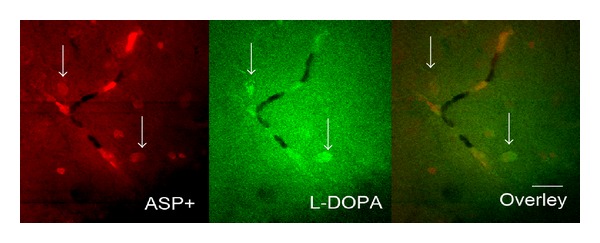
Double staining of hippocampal brain slice with l-DOPA and ASP^+^ (fluorescent substrate for transporters present in astrocytes and some other cells). Uptake revealed with the Falck-Hillarp method (see. Section 2). Astrocytes take up both l-DOPA and ASP^+^ (indicated with arrows). Scale: 20 *μ*m.

Brain slices were incubated in oxygenated ACSF containing 10 *μ*M l-DOPA and 1 *μ*M ASP^+^ (pH 7.4) for 30 min at room temperature. Slices were then dried and processed according to the modified Falck-Hillarp method (see Section 2). Using both filter sets for l-DOPA and ASP^+^ on the same preparation, we found a cooccurrence of ASP^+^ and l-DOPA accumulation in the same cells, including astrocytes and their endfeet on blood vessels (Figure 3). These data confirmed that both substrates were taken up by astrocytes in hippocampal slice preparations.

#### 3.3.2. **l-DOPA** Uptake by Astrocytes

**Figure 4:**
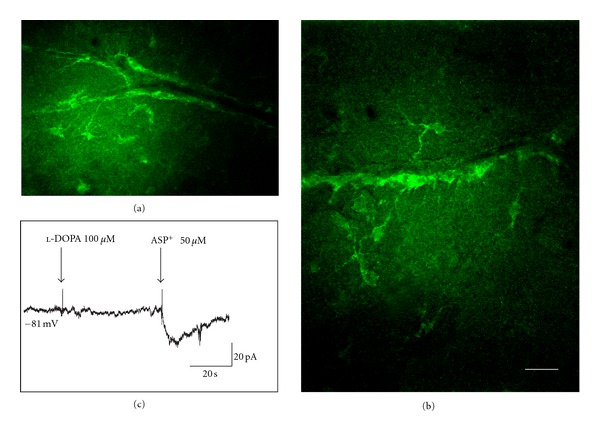
l-DOPA uptake by astrocytes and their endfeet on brain capillaries. (a) and (b) Uptake of l-DOPA as revealed by the Falck-Hillarp method is concentrated in astrocyte cell body, astrocyte processes that project to the vessels and in the endfeet touching the vessel wall. Note that the capillary vessel walls are completely enwrapped by the endfeet. (c) Current responses of an astrocyte during puff-application of l-DOPA (100 *μ*M) or ASP^+^ (50 *μ*M). Note that l-DOPA application did not elicit a current response, while ASP^+^ elicited an inward current. Scale: 20 *μ*m.

We used the Falck-Hillarp method to determine that l-DOPA uptake alone was more effective than in combination with ASP^+^, because ASP^+^, especially at concentrations greater than 1 *μ*M, interfered with l-DOPA uptake. Fluorescent astrocytic endfeet surround the hippocampal capillaries, and some astrocyte cell bodies and the processes that extend toward the capillaries were also clearly fluorescent (Figure 4, also see stacks in 3D supplementary material available online at doi:10.1155/2012/321406). Confocal images revealed that astrocytic endfeet cover the entire capillary wall without visible gaps (Figures 4(a) and 4(b)). These data suggest that astrocytes and their endfeet surrounding brain capillaries, as well as the process or processes that extend toward the vessels participate in l-DOPA uptake.

 Using live brain slices perfused with ACSF, we also patch-clamped astrocytes in voltage clamp mode, under similar conditions as used for studying ASP^+^ uptake (Figure 4(c)). The holding potential was maintained to keep zero current, and 100 *μ*M l-DOPA was puff-applied to the astrocyte, followed 100 s later with 50 *μ*M ASP^+^ similarly applied for comparison (applications marked by arrows). There was an obvious difference between the effects of ASP^+^ and l-DOPA on astrocyte currents: there was no elicited inward transporter current in the case of l-DOPA application, suggesting l-DOPA may enter the astrocyte via a different pathway or that it is transported by the same transporter, but in a different manner.

### 3.4. The Distribution of Monoamino Oxidase Type B (MAO-B) in Astrocytes and Capillaries

**Figure 5:**
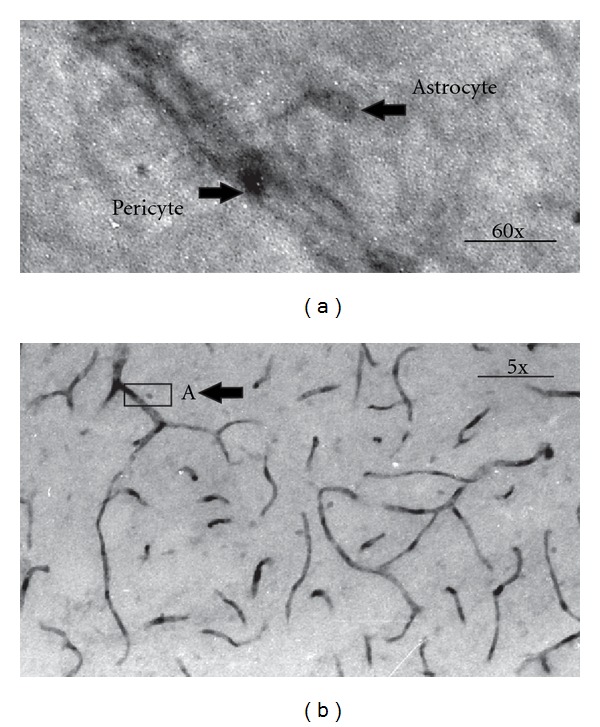
The pattern of MAO-B distribution in capillaries and astrocytes ((a) and (b)). (b) General pattern of MAO-B distribution in blood vessels and nearby astrocytes. Arrow indicates the insert. (a) Insert from (b), revealing at larger magnification a part of a capillary with a pericyte and an astrocyte cell body (both shown by arrows) with an endfoot process extending toward the vessel. Scale: 10 *μ*m in (a), 100 *μ*m, in (b).

 The presence of MAO-B in astrocytes [27–29] and in blood vessels with pericytes [18] is well established. Blood vessels with dark staining pericytes can be easily identified (Figure 5(b)) in our slices after visualization of MAO-B. In addition, stained astrocytes and their processes projecting towards the vessel can also be observed (Figure 5(a)). In these experiments, we were interested in determining if the pattern of MAO distribution resembles that of l-DOPA uptake. Generally, MAO-B was located in the pericytes and in the astrocyte cell bodies, the astrocyte processes projecting to the vessel and in the astrocytic endfeet. This was similar to the l-DOPA accumulation pattern suggesting that l-DOPA may be destroyed in these cells after uptake, at least partially.

## 4. Discussion and Conclusions

 Our experiments have confirmed that astrocytes, characterized as electrically passive astrocytes by patch-clamp methods, readily take up l-DOPA, as well as the fluorescent monoamine analog ASP^+^ in hippocampal slices. l-DOPA accumulation in astrocyte endfeet, in the astrocyte processes that project to the capillaries and in cell bodies suggests that the gliovascular interface may play an important role in dopamine precursor uptake, previously overlooked. Passive astrocytes are known to form an astrocytic network [1, 22, 30], in which astrocytes are interconnected with each other via gap junctions, and substances such as l-DOPA may be taken up near the blood vessels and may spread through this network.

 Participation of astroglia in l-DOPA uptake is very intriguing because astrocytes have definitely been shown to contribute to l-DOPA-to-dopamine conversion. Dopamine was detected in both rat and mouse cultured astrocytes after a 30 min incubation with l-DOPA, indicating the existence of aromatic l-amino acid decarboxylase [10, 31]. Also, aromatic l-amino acid decarboxylase mRNA was detected in primary cultures of astrocytes, and Western immunoblots demonstrated aromatic l-amino acid decarboxylase expression in astrocytes [32]. Interestingly, just 15 seconds after carotid injection of 3 mM/mL l-DOPA half of it was already decarboxylated to dopamine [17]. This occurred relatively uniformly and similarly in different parts of the brain and only about 10% more effectively in brain regions with pronounced dopaminergic and serotoninergic innervation [17]. The authors credited this decarboxylation to the endothelium cells, probably because previously aromatic l-amino acid decarboxylase activity was demonstrated in blood vessels and was attributed to the endothelium [16]. The close relationship between astrocytic endfeet and brain vessels was not well appreciated at that time. Even a very conservative estimate of the brain capillary-astrocyte contribution to decarboxylation of l-DOPA can account for at least 12% of all the aromatic l-amino acid decarboxylase activity after a double dopaminergic and serotoninergic chemical lesion [33].

 Uptake of ASP^+^ by astrocytes is very interesting because the well-known nonfluorescent analog and neurotoxic agent, 1-methyl-4-phenylpyridinium (MPP^+^), is probably transported into astrocytes by the same transporter. Astrocytes produce MPP^+^ from its precursor, MPTP [34], and MPP^+^ reverse transport has been shown to contribute to MPP^+^-related dopamine neuronal death [35]. It was also shown that organic cation transporters on the astrocytes adjacent to dopamine neurons contribute to damage by bidirectionally regulating the local bioavailability of the MPP^+^ and other similar toxic species [36]. ASP^+^, being a monoamine analog, is partly transported by intracellular transport to mitochondria where MAO is situated and concentrated [37]. It is known that MPP^+^ produces significant damage to mitochondria, what about ASP^+^?

In some atypical forms of Parkinsonism, the glial cells have been shown to malfunction due to other disease processes. This highlights the involvement of astrocytes in l-DOPA uptake and metabolism. One of clinical implications of glial l-DOPA processing may be the Parkinsonism manifested in multiple-system atrophy (MSA) when glial function is compromised [38, 39]. To what extent astrocytes are involved in MSA is unknown, but l-DOPA fails to improve the parkinsonian symptoms of most MSA patients. Poor response to l-DOPA has been suggested as a possible element in the differential diagnosis of MSA from Parkinson's disease [39].

Fast l-DOPA uptake and conversion to dopamine by glial cells can also contribute to pulsatile changes of dopamine levels in cerebrospinal fluid after oral l-DOPA pill consumption, as well as oxidation of excess monoamines or methylation of l-DOPA to its main metabolites (ex. 3-O-methyldopa). These concentration spikes are associated with the development of motor complications and even the onset of psychosis [40].

It is important to note that MAO-B is considered to be the main pathway for the dopamine degradation in astrocytes [28, 41]. For this reason we used MAO-B staining to visualize glia. But older data about the MAO-A abundance in glia [42] and newer data about the therapeutic potential of mixed nonselective MAOA/B monoamine oxidase inhibitors [43] raise the possibility that glial MAO-A may also participate in dopamine degradation, in particular, when dopamine levels are in excess.

 Glial turnover of the l-DOPA metabolite of dopamine via MAO-B is a further source of increased synthesis of free radicals [40], producing gliosis and compensatory changes in protein expression in astrocytes. For example, production of endothelial growth factor (EGF) is upregulated by l-DOPA in the Parkinsonian brain. EGF is expressed mainly by astrocyte processes and astrocyte endfeet on blood vessels and overexpression can lead to abnormal vessel density and ultimately to the development of dyskinesia [44]. Earlier it was proposed that endothelial proliferation after exposure to high concentrations of l-DOPA can lead to breach in the blood-brain barrier (BBB) that itself can be the cause of dyskinesia [45]. This finding is controversial because a later study using in vivo neuroimaging demonstrated that the BBB is intact after l-DOPA-induced dyskinesias in parkinsonian animals [46]. Also, Müller and coauthors [47] showed that the level of 3-O-methyldopa does not affect significantly l-DOPA pharmacokinetics and motor responses in patients and they concluded that the BBB was not affected.

Our aim was to demonstrate that astrocytes, as well as pericytes and endothelial cells, are able to take up l-DOPA, convert it to dopamine, and also have all necessary oxidative machinery to metabolize dopamine. They can release dopamine using different mechanisms; for example, by reverse transport. MAO-B blockers may be able to avert this astrocyte-mediated dopamine destruction. There are still a lot of open questions. It was shown that astrocytes release dopamine [10, 31], but it is not known which transporter is releasing dopamine from astrocytes. Could it be the organic cation transporter? How important is the glial participation in l-DOPA uptake and conversion? Does glial uptake of l-DOPA change during the progression of Parkinson's disease?

 Finally, we can conclude that (i) astrocytes participate in l-DOPA uptake and metabolism via the gliovascular interface and, therefore, may play a key role in regulating brain dopamine, (ii) the role of astroglia in l-DOPA uptake and processing must be reconsidered specifically in cases of Parkinson's disease, and (iii) our data also suggest that different transporter mechanisms may exist for monoamines and l-DOPA uptake by astrocytes.

## Supplementary Material

PowerPoint presentation of Figures from this article, some of them in 3D, as it was reported at the Annual Society for Neuroscience (SFN), 2011.
