# Supplementary material for: L-DOPA Uptake in Astrocytic Endfeet Enwrapping Blood Vessels in Rat Brain
Source: Parkinsons Dis. 2012 Jul 24;2012:321406. doi: 10.1155/2012/321406 (PMC3409556; doi:10.1155/2012/321406)
Supplement: Supplementary file 1 — PowerPoint presentation of Figures from this article, some of them in 3D, as it was reported at the Annual Society for Neuroscience (SFN), 2011. [file 321406.f1.ppt]

## Slide 1
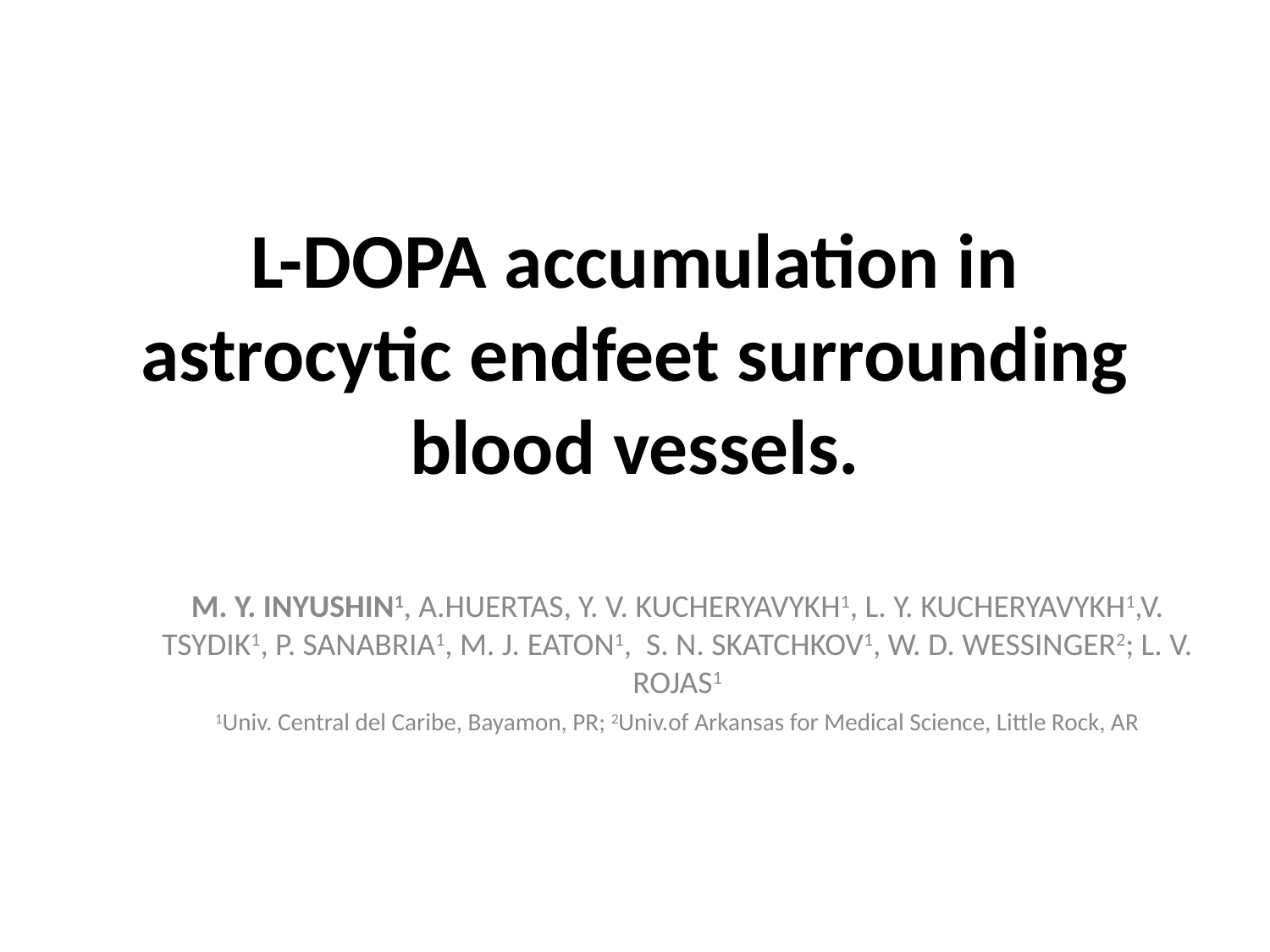

# L-DOPA accumulation in astrocytic endfeet surrounding blood vessels.
M. Y. INYUSHIN1, A.HUERTAS, Y. V. KUCHERYAVYKH1, L. Y. KUCHERYAVYKH1,V. TSYDIK1, P. SANABRIA1, M. J. EATON1, S. N. SKATCHKOV1, W. D. WESSINGER2; L. V. ROJAS1
1Univ. Central del Caribe, Bayamon, PR; 2Univ.of Arkansas for Medical Science, Little Rock, AR

## Slide 2
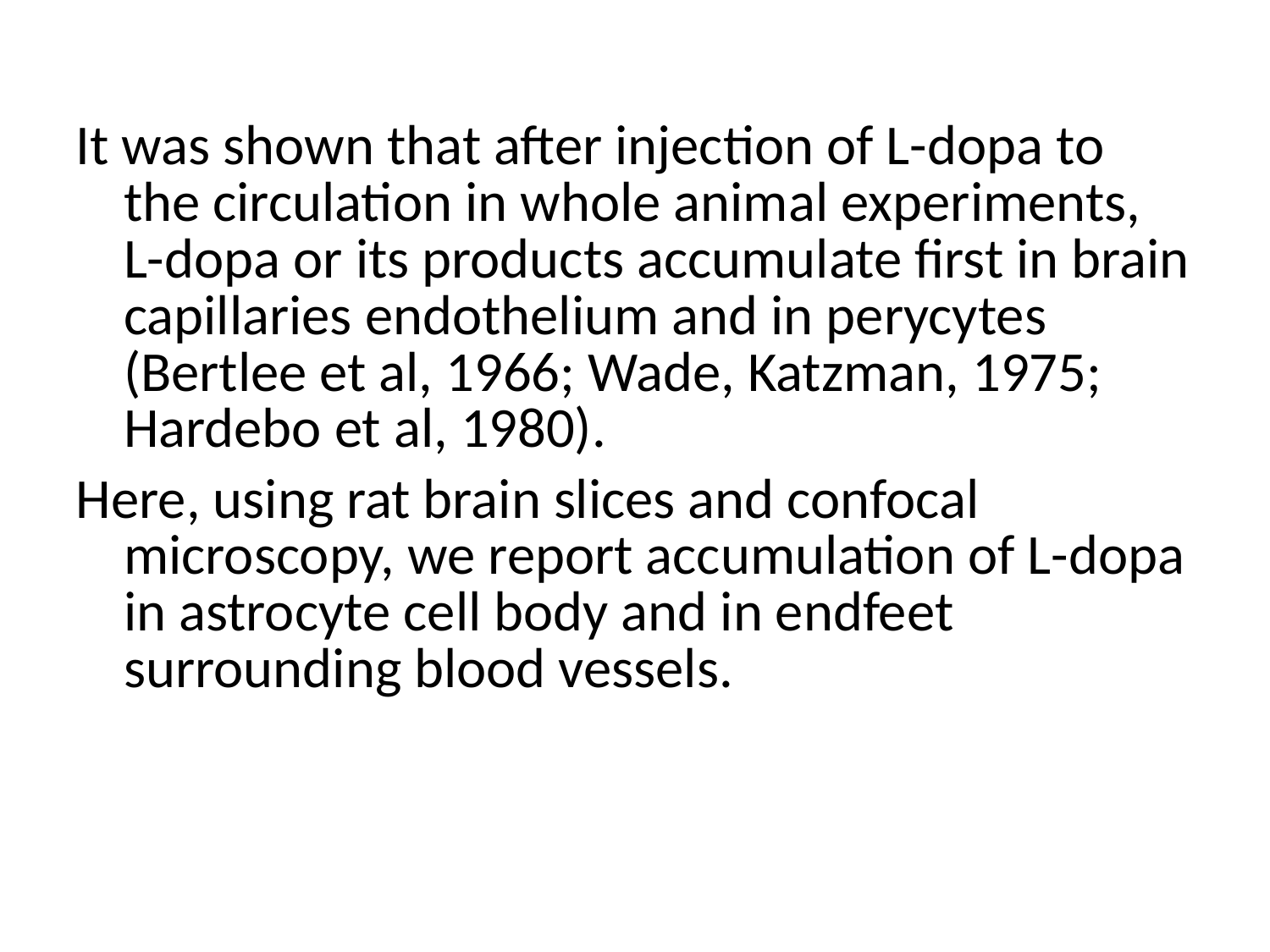

It was shown that after injection of L-dopa to the circulation in whole animal experiments, L-dopa or its products accumulate first in brain capillaries endothelium and in perycytes (Bertlee et al, 1966; Wade, Katzman, 1975; Hardebo et al, 1980).
Here, using rat brain slices and confocal microscopy, we report accumulation of L-dopa in astrocyte cell body and in endfeet surrounding blood vessels.

## Slide 3
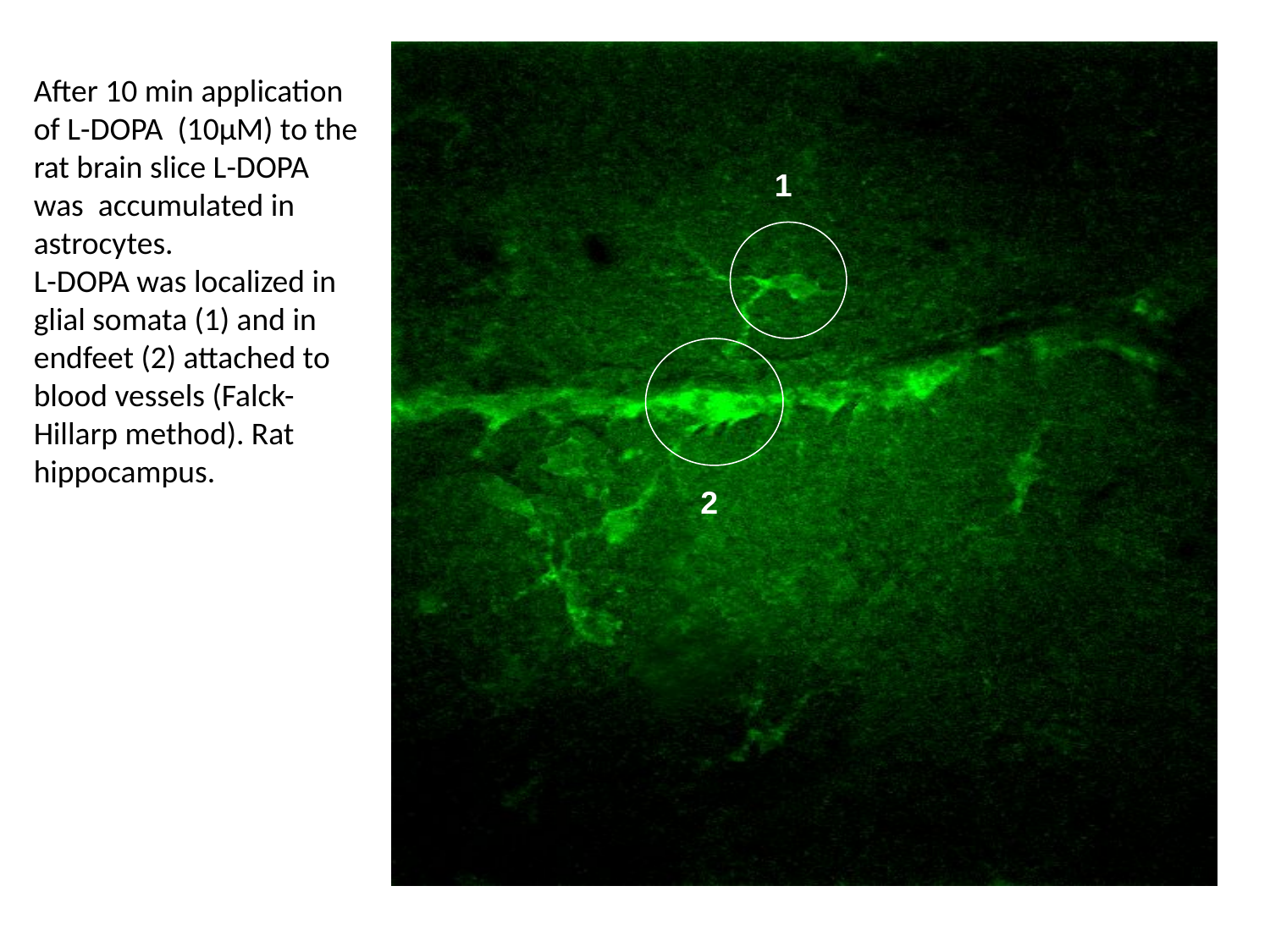

After 10 min application of L-DOPA (10µM) to the rat brain slice L-DOPA was accumulated in astrocytes.
L-DOPA was localized in glial somata (1) and in endfeet (2) attached to blood vessels (Falck-Hillarp method). Rat hippocampus.
1
2

## Slide 4
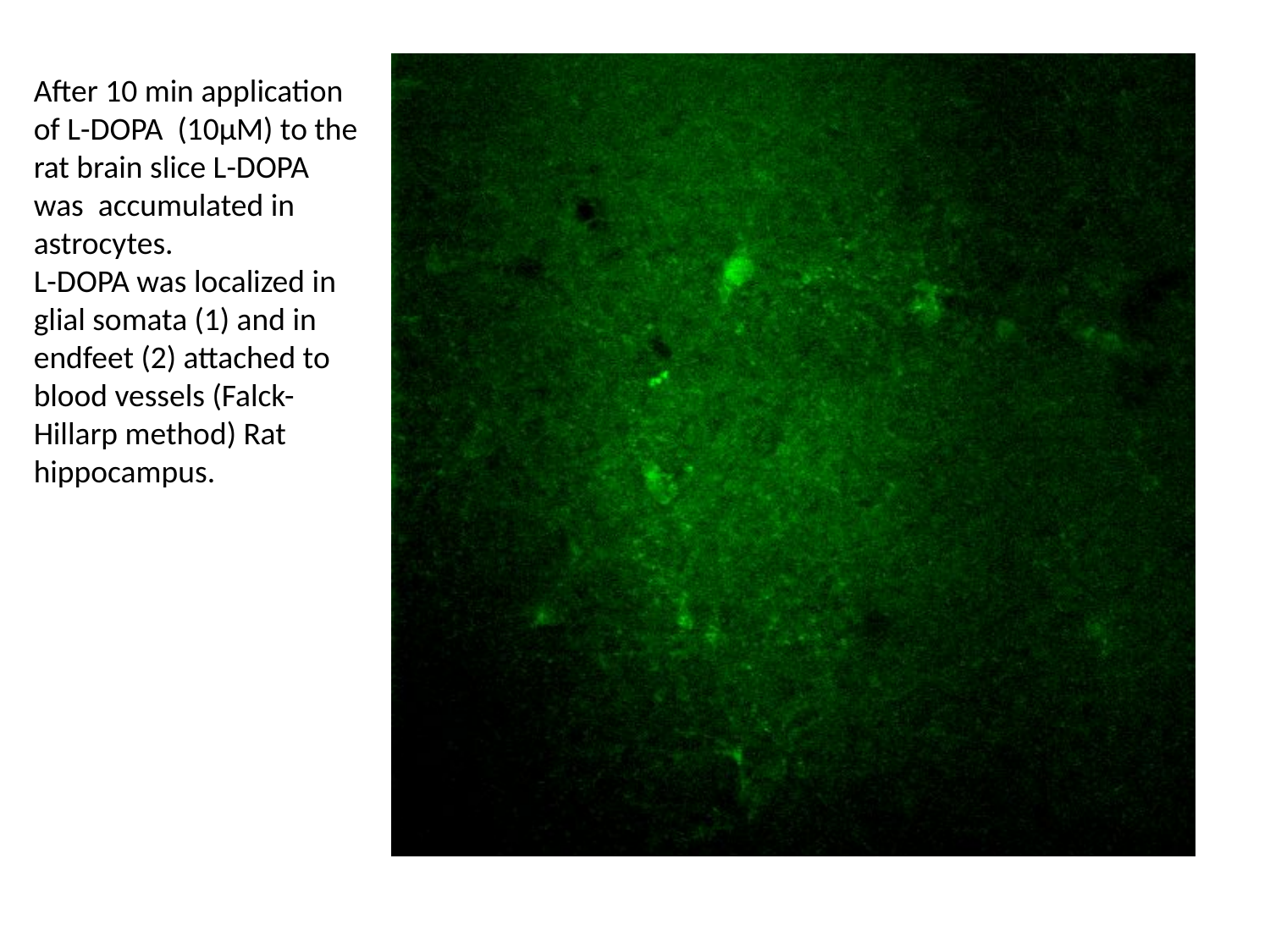

After 10 min application of L-DOPA (10µM) to the rat brain slice L-DOPA was accumulated in astrocytes.
L-DOPA was localized in glial somata (1) and in endfeet (2) attached to blood vessels (Falck-Hillarp method) Rat hippocampus.

## Slide 5
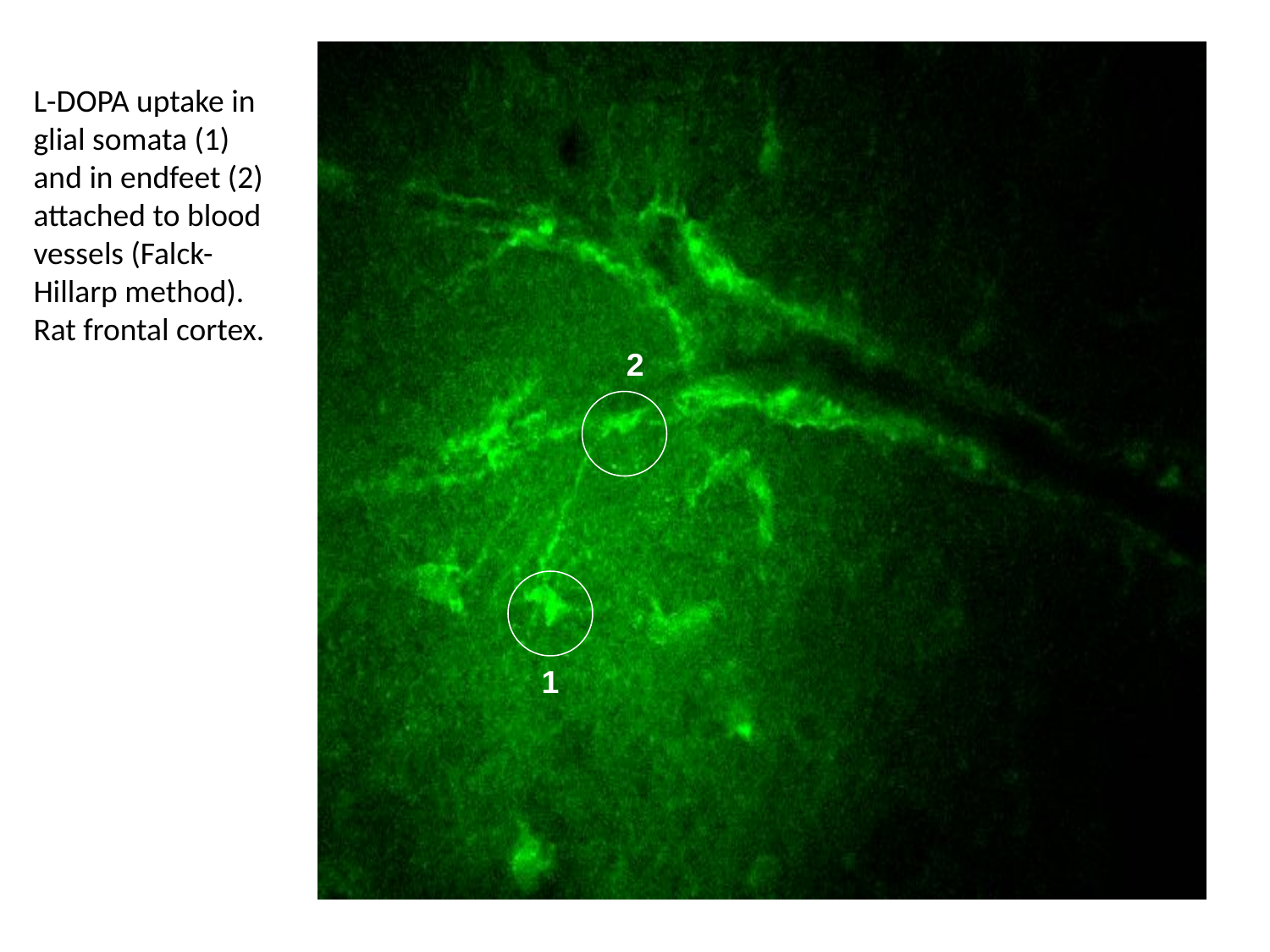

L-DOPA uptake in glial somata (1) and in endfeet (2) attached to blood vessels (Falck-Hillarp method). Rat frontal cortex.
2
1

## Slide 6
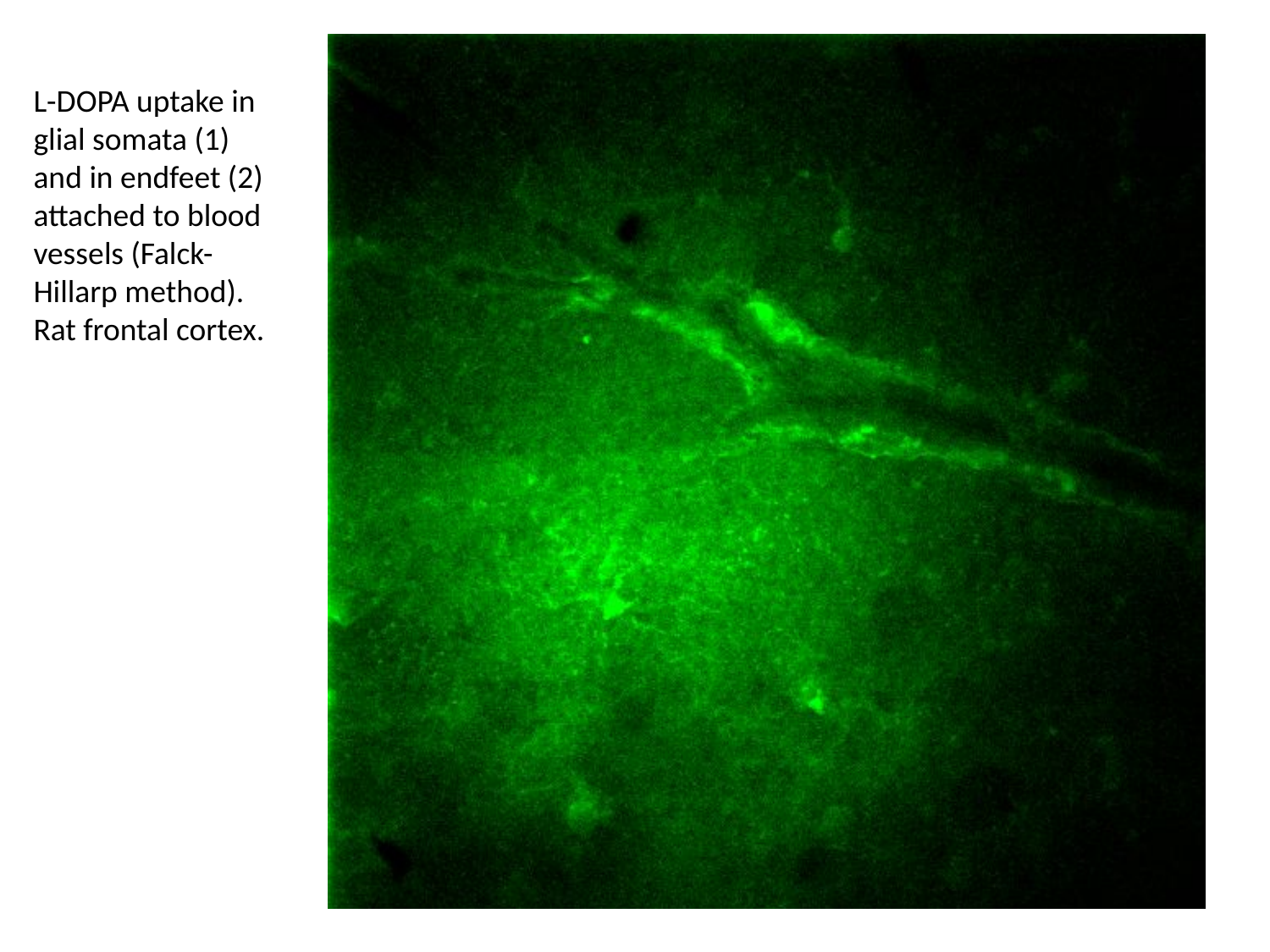

L-DOPA uptake in glial somata (1) and in endfeet (2) attached to blood vessels (Falck-Hillarp method). Rat frontal cortex.

## Slide 7
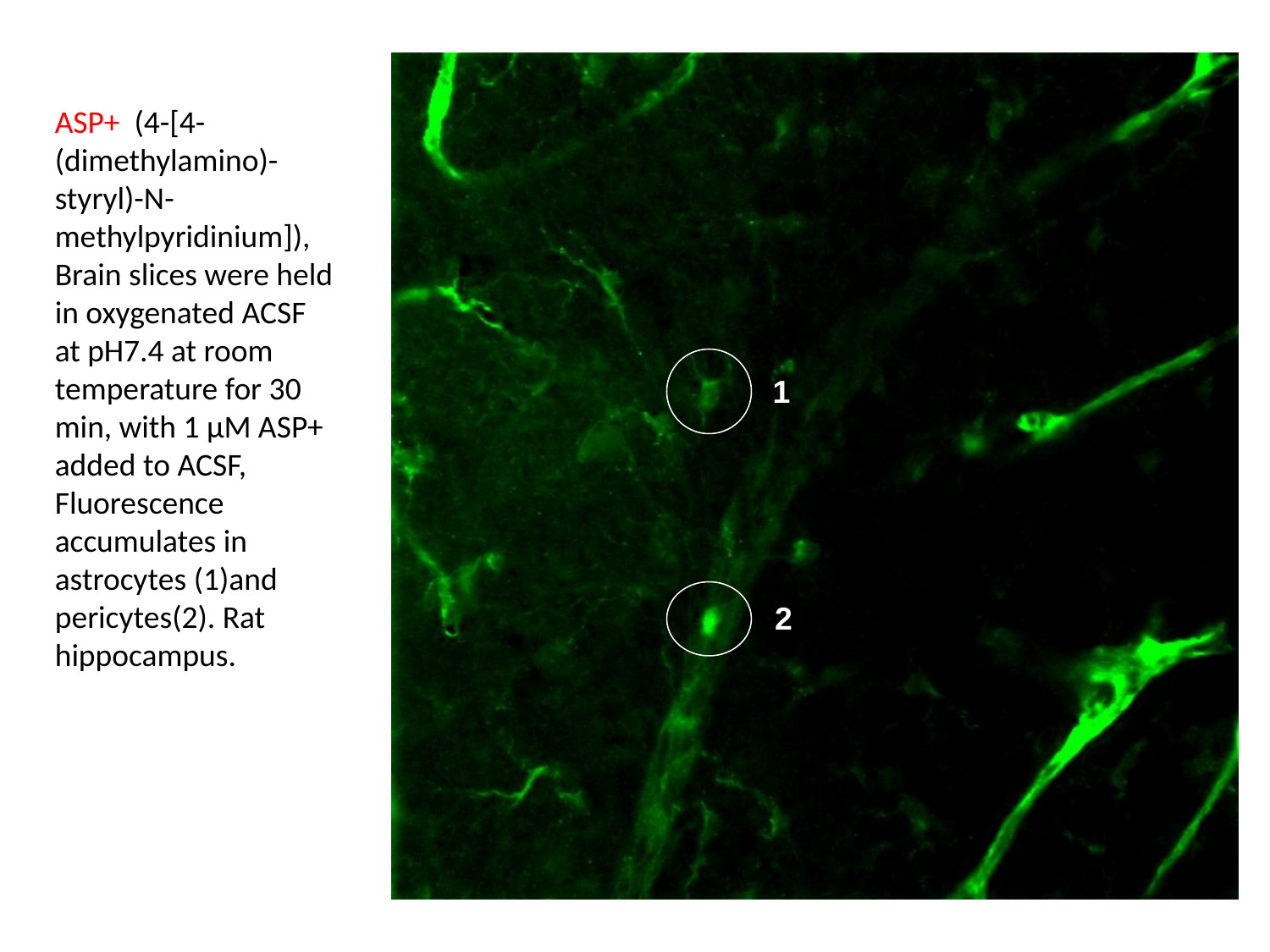

ASP+ (4-[4-(dimethylamino)-styryl)-N-methylpyridinium]),
Brain slices were held in oxygenated ACSF at pH7.4 at room temperature for 30 min, with 1 µM ASP+ added to ACSF, Fluorescence accumulates in astrocytes (1)and pericytes(2). Rat hippocampus.
1
2

## Slide 8
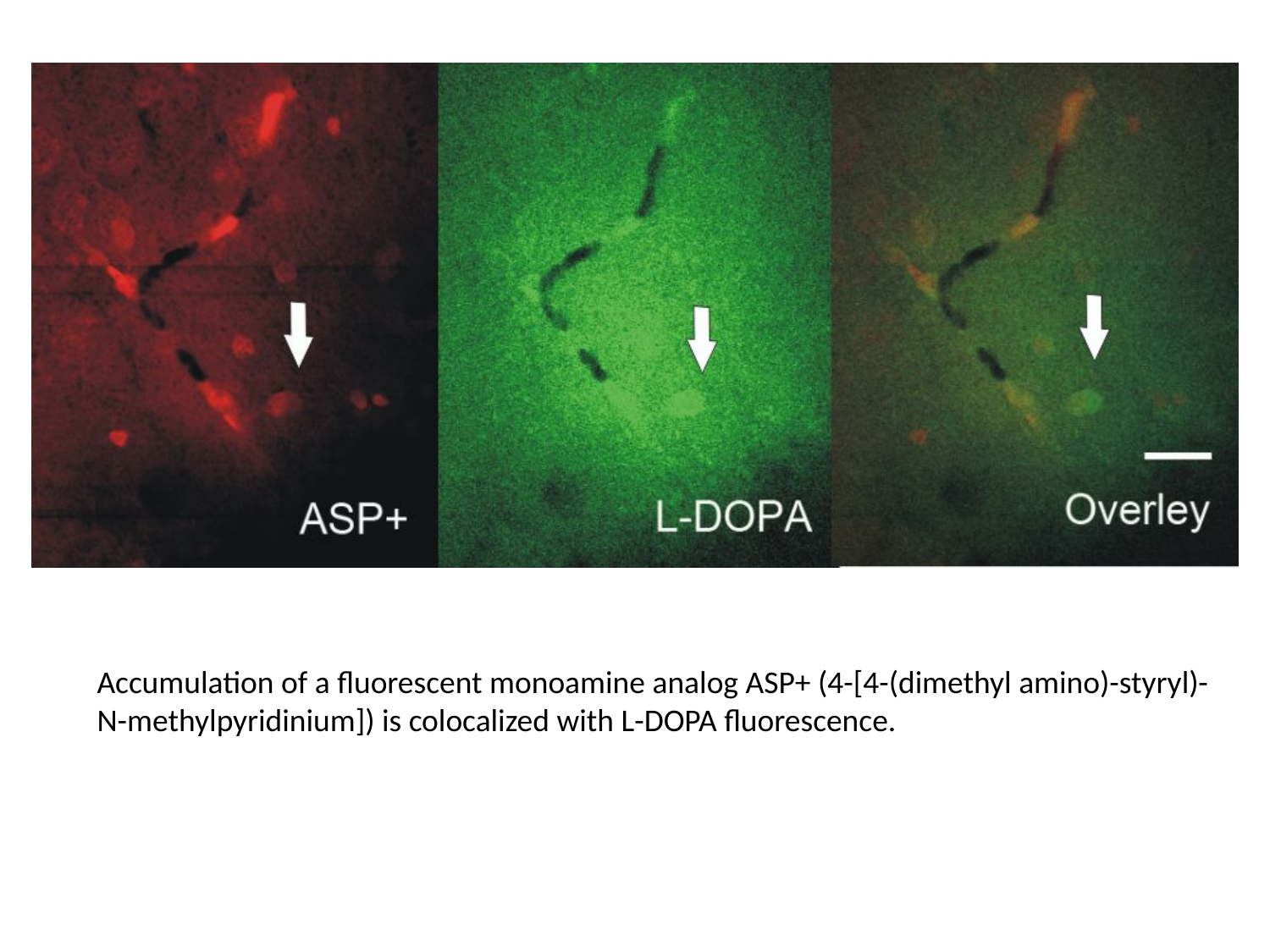

Accumulation of a fluorescent monoamine analog ASP+ (4-[4-(dimethyl amino)-styryl)-N-methylpyridinium]) is colocalized with L-DOPA fluorescence.

## Slide 9
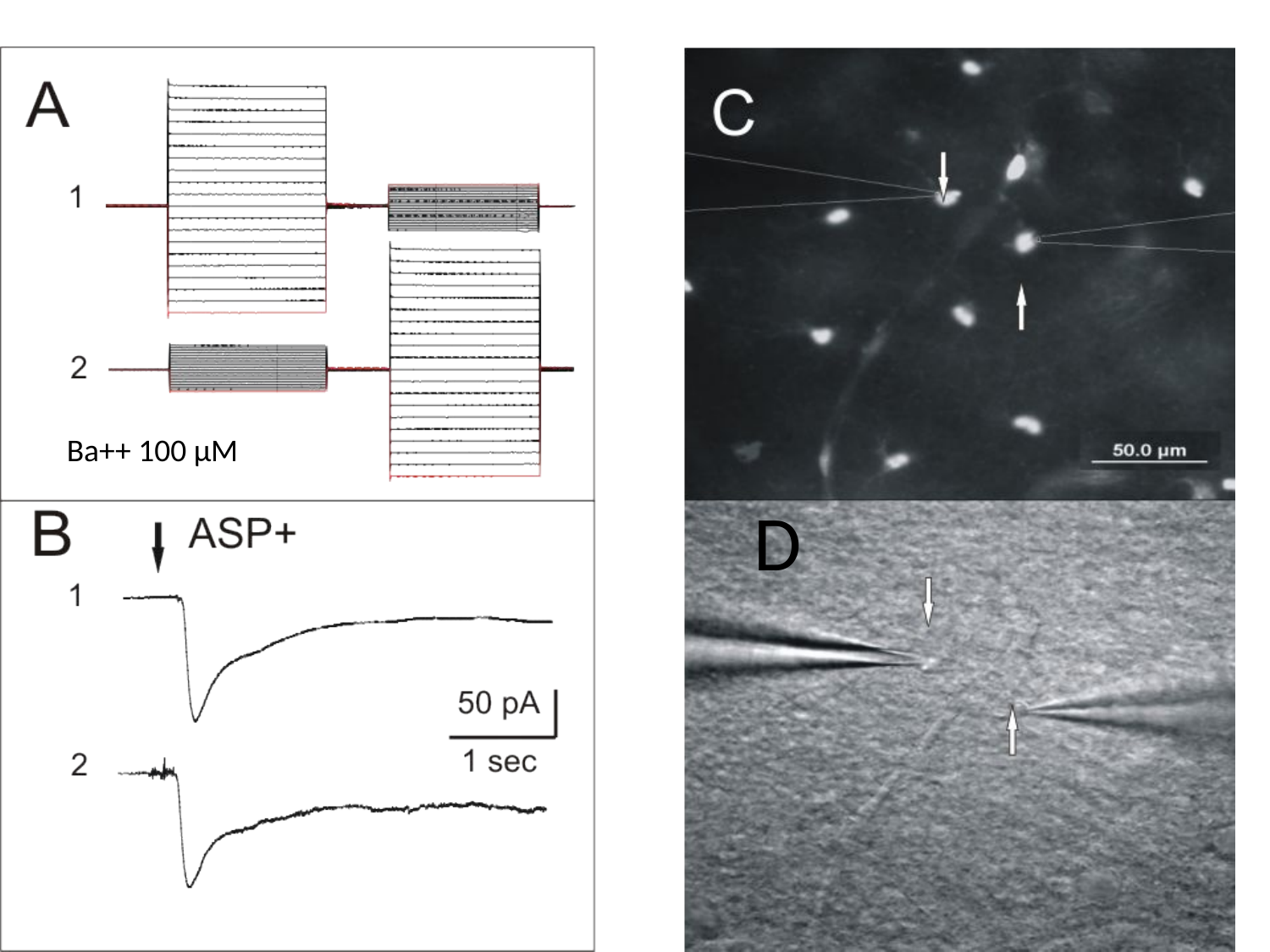

Ba++ 100 µM
D

## Slide 10
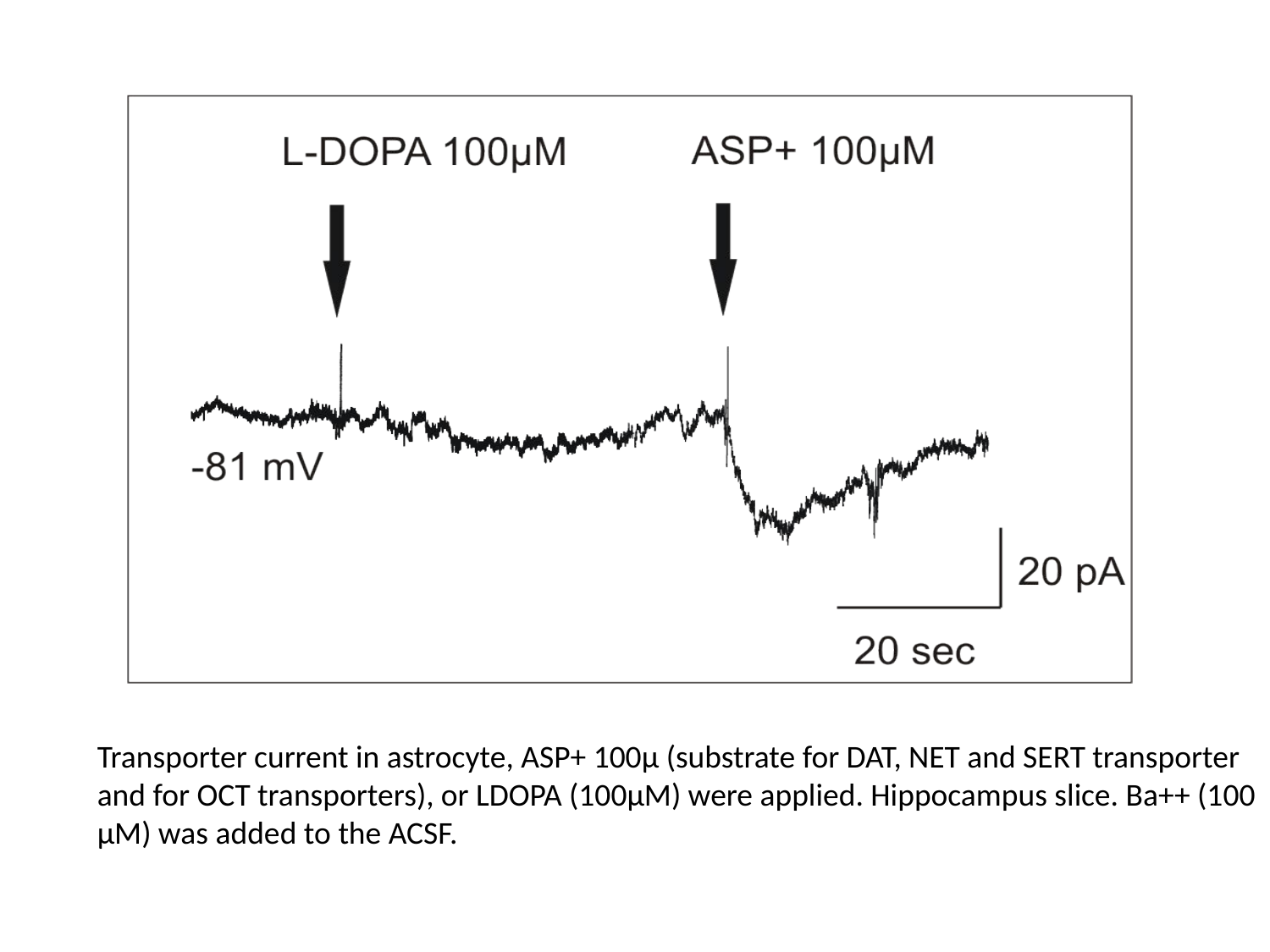

Transporter current in astrocyte, ASP+ 100µ (substrate for DAT, NET and SERT transporter
and for OCT transporters), or LDOPA (100µM) were applied. Hippocampus slice. Ba++ (100 µM) was added to the ACSF.

## Slide 11
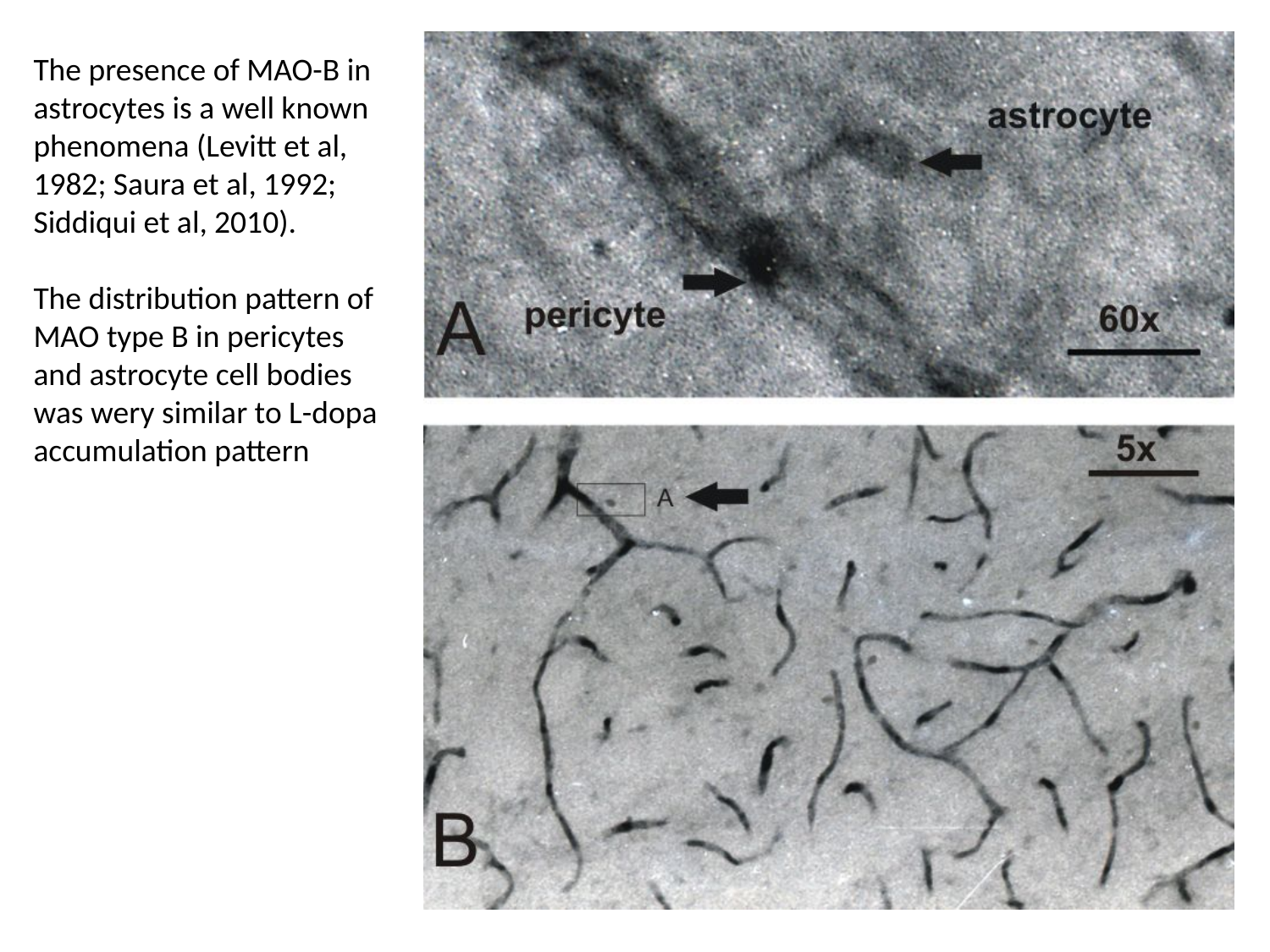

The presence of MAO-B in astrocytes is a well known phenomena (Levitt et al, 1982; Saura et al, 1992; Siddiqui et al, 2010).
The distribution pattern of MAO type B in pericytes and astrocyte cell bodies was wery similar to L-dopa accumulation pattern

## Slide 12
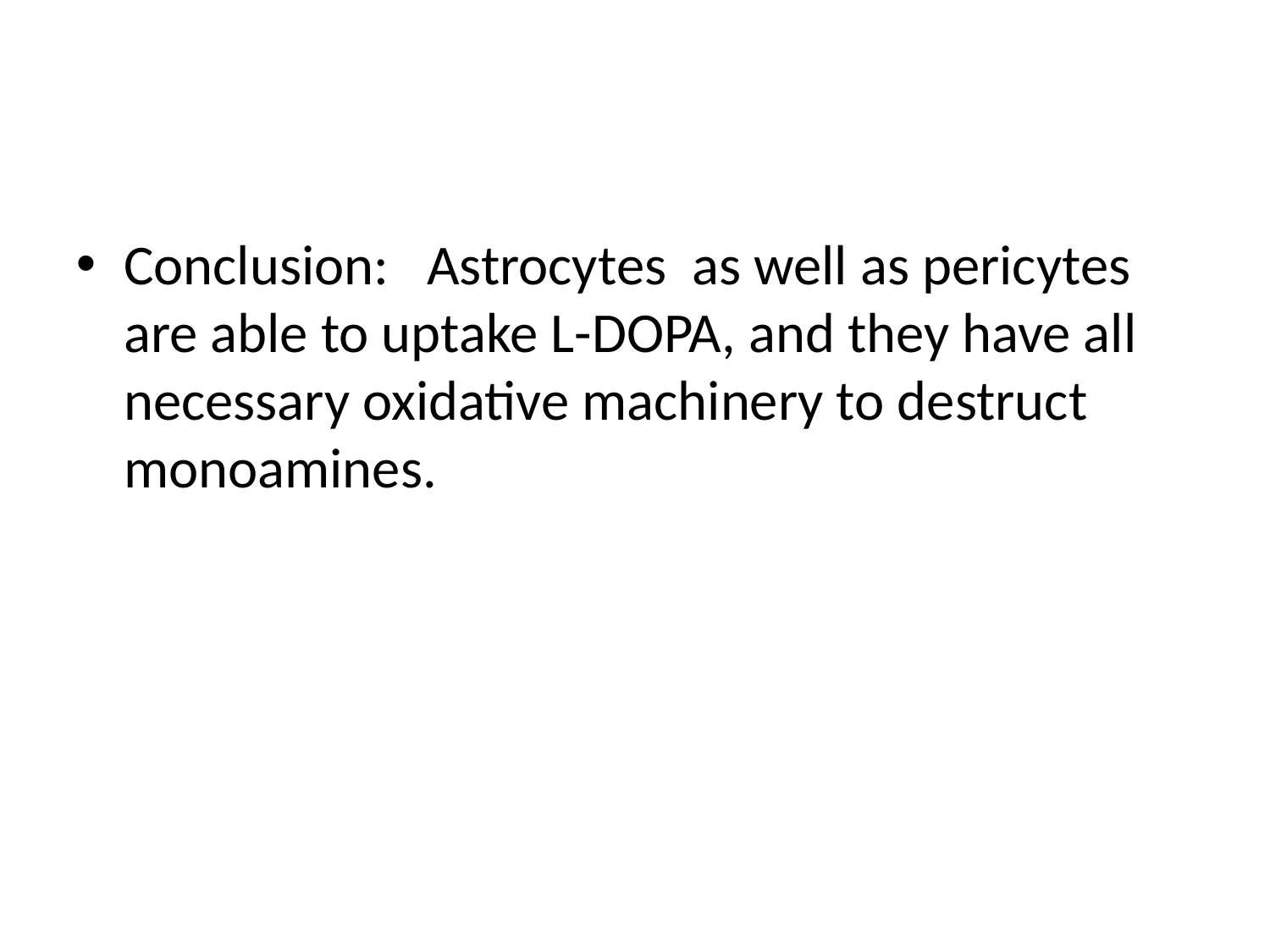

Conclusion: Astrocytes as well as pericytes are able to uptake L-DOPA, and they have all necessary oxidative machinery to destruct monoamines.

## Slide 13
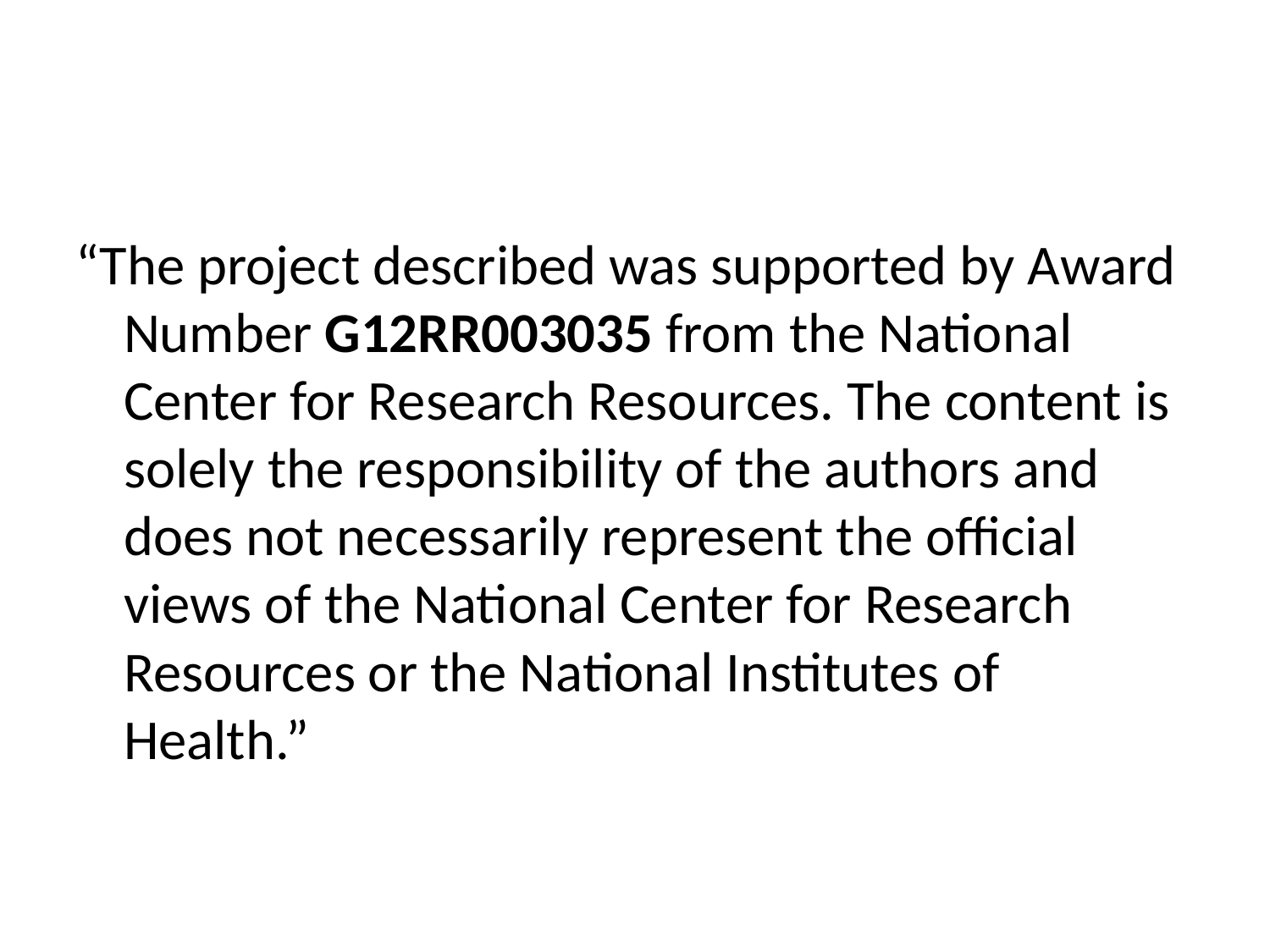

“The project described was supported by Award Number G12RR003035 from the National Center for Research Resources. The content is solely the responsibility of the authors and does not necessarily represent the official views of the National Center for Research Resources or the National Institutes of Health.”

## Slide 14
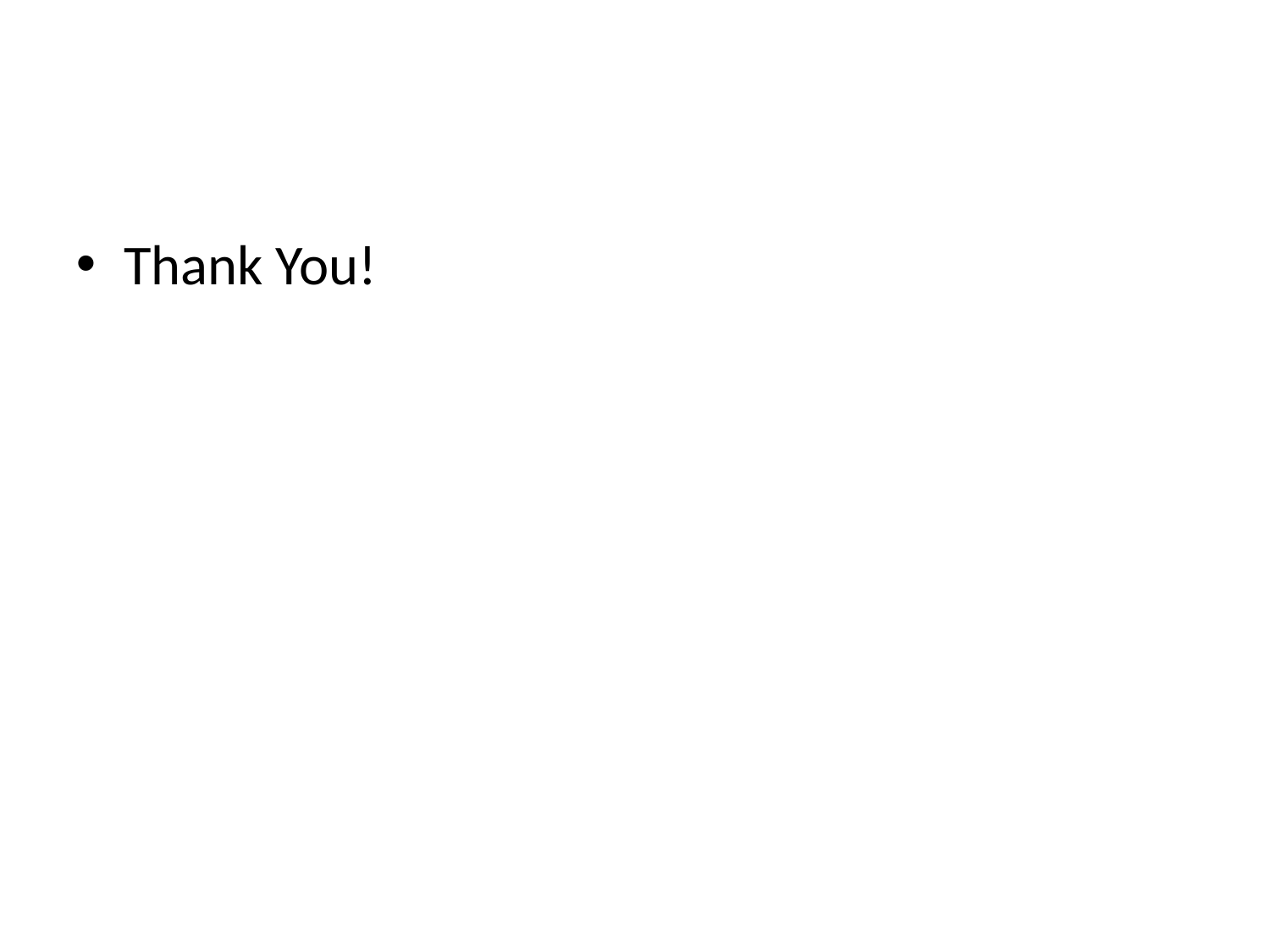

Thank You!

## Slide 15
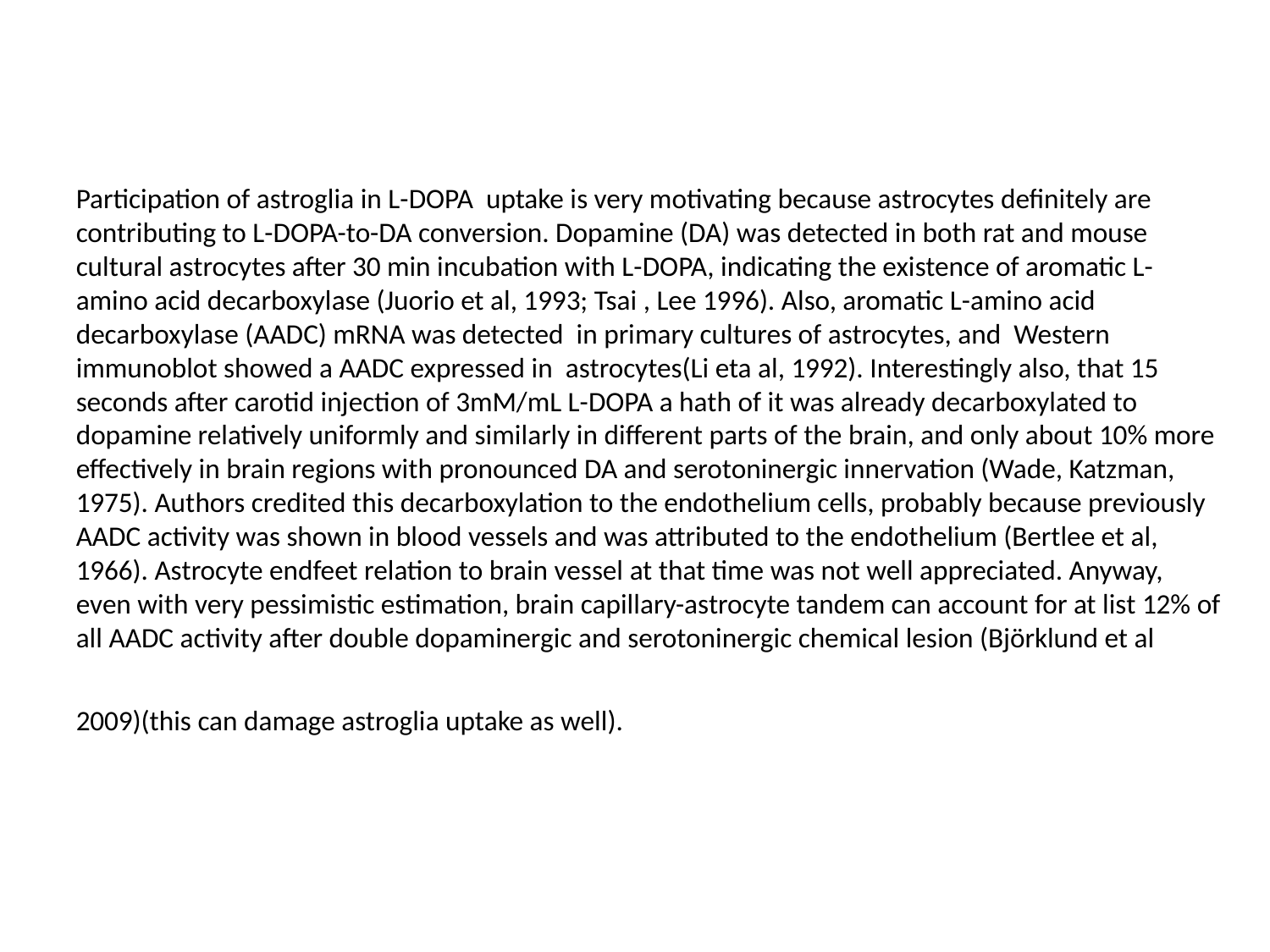

Participation of astroglia in L-DOPA uptake is very motivating because astrocytes definitely are contributing to L-DOPA-to-DA conversion. Dopamine (DA) was detected in both rat and mouse cultural astrocytes after 30 min incubation with L-DOPA, indicating the existence of aromatic L-amino acid decarboxylase (Juorio et al, 1993; Tsai , Lee 1996). Also, aromatic L-amino acid decarboxylase (AADC) mRNA was detected in primary cultures of astrocytes, and Western immunoblot showed a AADC expressed in astrocytes(Li eta al, 1992). Interestingly also, that 15 seconds after carotid injection of 3mM/mL L-DOPA a hath of it was already decarboxylated to dopamine relatively uniformly and similarly in different parts of the brain, and only about 10% more effectively in brain regions with pronounced DA and serotoninergic innervation (Wade, Katzman, 1975). Authors credited this decarboxylation to the endothelium cells, probably because previously AADC activity was shown in blood vessels and was attributed to the endothelium (Bertlee et al, 1966). Astrocyte endfeet relation to brain vessel at that time was not well appreciated. Anyway, even with very pessimistic estimation, brain capillary-astrocyte tandem can account for at list 12% of all AADC activity after double dopaminergic and serotoninergic chemical lesion (Björklund et al 2009)(this can damage astroglia uptake as well).
